# The extent of *Ds1* transposon to enrich transcriptomes and proteomes by exonization

**DOI:** 10.1186/1999-3110-54-14

**Published:** 2013-08-21

**Authors:** Yuh-Chyang Charng, Li-yu Daisy Liu

**Affiliations:** Department of Agronomy, National Taiwan University, No. 1 Sec. 4 Roosevelt Rd, Taipei, Taiwan, Republic of China

**Keywords:** Alternative splicing, *Ds1* transposon, Exonization, Nonsense-mediated decay pathway

## Abstract

**Background:**

Exonization is an event which an intronic transposed element (TE) provides splice sites and leads to alternatively spliced cassette exons. Without disrupting of the inserted gene’s function, TEs can expand the proteome diversity by adding the splice variant that encodes a different, yet functional protein. Previously, we found that the main contribution of *Ds* exonization for gene divergence is not providing genetic messages but incorporating the intron sequences with different reading frame patterns to enrich the plant proteome. *Ds1*, another member of *Ac*/*Ds* transposon system, differs from *Ds* by providing 3 splice donor sites and 2 acceptor sites for alternative splicing, which may greatly increase the extent for proteome expansion.

**Results:**

In this study, we performed a genome-wide survey of *Ds1* exonization events to assess its extent to enrich proteomes in plants. Each *Ds1* insertion yielded 11 transcript isoforms by integrating the splice donor and/or acceptor sites, which composed a bulk of all exonized transcript orthologs from the dicot *Arabidopsis thaliana* and the monocot *Oryza sativa* (rice). The exonized transcripts were analyzed by the locations of the termination codon (PTC) and the putative targets for the nonsense-mediated decay (NMD) pathway were then excluded. Compared with the *Ds* element, *Ds1* harbors more contents of non-NMD transcripts for protein isoforms.

**Conclusions:**

The contribution of *Ds1* exonization for gene divergence is incorporating the intron sequences with different reading frame patterns to enrich the plant proteome. All these simulation results direct new experimental analysis at the molecular level.

**Electronic supplementary material:**

The online version of this article (doi:10.1186/1999-3110-54-14) contains supplementary material, which is available to authorized users.

## Background

Insertion of transposed elements (TEs) within eukaryotic genes is thought to be an important contributor to evolution and speciation (Sela et al., 2010). A well-known effect of TEs is to disrupt the function of the inserted gene, mostly in exons. However, TEs inserted into intronic sequences may not disrupt the target gene but, by alternative splicing (AS) and exonization, alter the regular splicing pattern of a pre-mRNA and result in the translation of new protein isoforms (Feschotte, 2008). With AS, the inserted TE interferes with the normal splicing of a gene’s transcribed region. With exonization, the inserted TE offers cryptic splice sites incorporated (exonized) as an alternative exon. While the prevailing original splice variant maintains functionality, the additional sequence, free from selection pressure, evolves a new function or eventually vanishes. If the new splice variant is advantageous, selection might operate to optimize the new splice sites and consequently increase the proportion of the alternative splice variant (Schmitz and Brosius, 2011).

Even in the absence of TE insertions, AS is a widespread phenomenon in higher eukaryotes. Eukaryotes can produce different mRNAs from a single gene transcript through the process of AS. More than 60% of human genes and around 20–30% of plant genes undergo AS (Campbell et al., 2006; Kim et al., 2007; Wang and Brendel, 2006). Yet, the extent to which AS leads to functional protein isoforms and to proteome expansion at large is still in dispute. (Severing et al. 2009) performed a detailed comparison of AS events in alternative spliced orthologs from the dicot *Arabidopsis thaliana* and the monocot *Oryza sativa* (rice) and revealed that AS has a limited role in functional expansion of the plant proteome. This conclusion was based on the ability of AS to add or delete functional protein domains. Those AS events, which result in small stretches of amino acids and therefore modify protein domains, need further structural and experimental analyses.

Unlike AS, exonization may insert portion(s) of the TE transcripts into the target gene and alter the reading frames to enrich the complexity of proteomes. Recent studies of exonization have mostly involved mammalian TEs *in silico* (Levy et al., 2008; Mersch et al., 2007; Mola et al., 2007; Sela et al., 2007). Many results also provided mechanistic insights into the process of exonization, especially 5’ and 3’ splice sites (i.e., splice donor/acceptor) formation in Alu exons (Krull et al., 2007; Lev-Maor et al., 2007; Ram et al., 2008; Sorek et al., 2004). In plants, we had assessed the ability of a TE to provide splice/acceptor sites by inserting a mini *Ds* transposon into each intron of the *epsps* gene (Charng et al., 2008; Huang et al., 2012) and found that *Ds* is biased in favor of providing splice donor sites from the beginning of the inserted *Ds* sequence. We also performed a genome-wide survey of *Ds* exonization to enrich transcriptomes and proteomes in plants (Liu and Charng, 2012) and found out up to 71% of the exonized transcripts were putative targets of the nonsense-mediated mRNA decay (NMD) pathway (Chang et al., 2007). Although the non-NMD exonized transcripts of *Ds* could be translated into abundant protein isoforms, it is interesting to study the extent to which the proteomes are triggered by another TE, *Ds1*, which can provide both donor and acceptor sites (Wessler, 1991).

**Figure 1:**
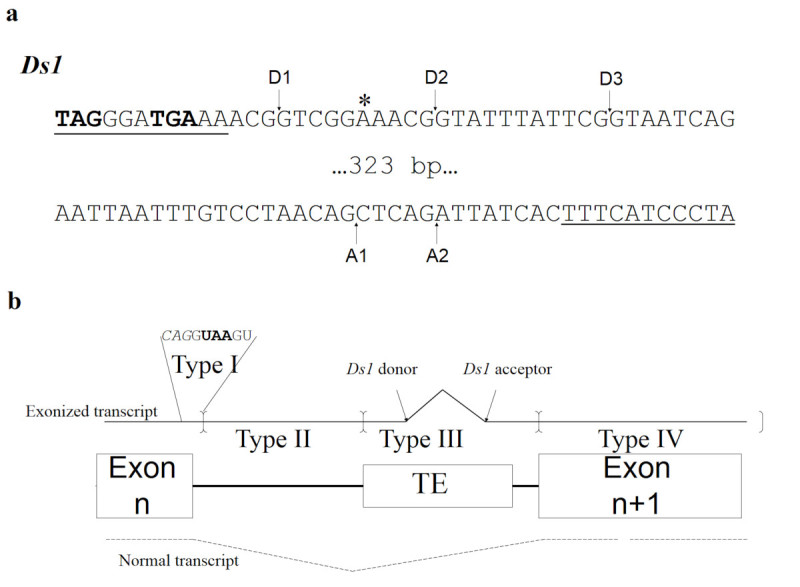
***Ds1*** **termini sequences and classification of exonized transcripts. a)** *Ds1* termini sequences providing splice donor/acceptor junction (arrows) and premature termination codons (PTCs) in exonized transcripts (bold). * indicates the nucleotide differs with *Ds* element, which harbors another PTC presenting as TAA. **b)** Classification of exonized transcripts (black line) according to location of the PTC. Normal transcript is shown as a dashed line. The corresponding DNAs of the TE-inserted target are shown in the centre. Black box indicates the unspliced intron. Exonization occurs by using the splice donor (arrow) of TE to join the upcoming exon. The existence and location of an in-frame PTC determines the type of the exonized transcripts, classified into 5 types. As an example, for type I, a PTC (UAA in bold) locates in the skipped exon/intron consensus: italics indicate exonic sequences. Brackets indicate the boundaries of PTC location for classification. Type V transcripts have no in-frame PTC until the end of the gene.

*Ds1* transposable element is a member of *Ac*/*Ds* transposon system. It can provide 5 splice sites for alternative splicing; 3 act as donors and 2 as acceptors. Unlike *Ds* containing splice sites in both forward and reverse forms (Huang et al., 2012), all splice sites of *Ds1* were observed in reverse pattern and locate less than 35 bp from both termini. Additionally, 2 donor sites (D1 and D2, Figure 1) of the *Ds1* were identical to those of the *Ds*, which were determined by RT-PCR experiments to be functional in tobacco and rice (Huang et al., 2012 and unpublished results).

In this study, we proposed a computational approach to genome-wide assess the role of *Ds1* exonization in plants. We simulated a bulk of all exonized transcript orthologs from the dicot *Arabidopsis thaliana* and the monocot *Oryza sativa* (rice). The resulting exoinzed transcripts were divided into 5 types by location of the termination codon (PTC) (Figure 1). The protein isoforms of the exonized transcripts bypassing the NMD pathway were further classified as C-terminal or interior variants to reveal the possible complexity of the proteome caused by *Ds1* exonization. Compared with *Ds* element, the *Ds1* harbors more possibilities for proteomes enrichment by combining the splice donors and acceptors for exonization. Therefore, the contribution of *Ds1* insertion to evolution may enrich the genome by incorporating the intronic sequences of the inserted genes into the exonized transcripts orthologs.

## Methods

### Data sources and exonized transcript construction

Arabidopsis and rice chromosome Genbank data and whole-genome sequences were downloaded from the NCBI database (http://www.ncbi.nlm.nih.gov/genomes/PLANTS/PlantList.html), and the amino acid coding region (CDS) for each gene was extracted. For rice, every gene has only one CDS record. However, for Arabidopsis, some genes have multiple CDS records, so we used only the first CDS record to avoid redundancy. Exonization was defined as an event in which a transcript variant was created with insertion of a TE in the intronic sequence of a gene. Therefore, we considered only genes that were completely sequenced and had at least 1 intron.

The construction of the exonized transcripts involved use of R (R Development Core Team, 2008). For each gene, we used a three-step procedure for every intron. Let a target gene, *G*, have *I* introns (and, of course, *I* + 1 exons), with the *i* th intron of length *n*_*i*_.

First, the sequence of the 512 bp *Ds1* was inserted in a forward or reverse direction after the *j* th nt (*j* = 0, …, *n*_*i*_) of the *i* th intron of *G*. The insertion we describe here means literally to insert the letters of *Ds1* after the *j* th nucleotide. This insertion was equivalent to a biological event of *Ds1* inserted at 8 bp before the assigned position. Biologically, the insertion of *Ds1* causes the duplication of 8 bp of *G* right after the insertion position, and the sequence of the *Ds1* starts at the 9th nt after the insertion position.

Second, we obtained all exonized sequences by recognizing appropriate splice donor/acceptor sites. From our previous observations, *Ds1* provides 3 donors (maximal 35 nucleotides from the donors-end) and 2 acceptors (24 nucleotides from the acceptors-end for the subsequent transcripts). This yielded 11 transcript isoforms for each *Ds1* insertion.

Finally, the exonized transcripts were constructed by joining the sequences. When *Ds1* only provides a donor, the exonized transcript combines the 1st to the *i* th exons, the first *j* nt of the *i* th intron, the *Ds1* sequences until the junction site, and the sequences of the (*i* + 1)th to the (*I* + 1)th exons. When *Ds1* only provides an acceptor, the exonized transcript combines the 1st to the *i* th exons, the *Ds1* sequences until the junction site, the *i* th intron starting from the (*j* + 1) nt to the end, and the sequences of the (*i* + 1)th to the (*I* + 1)th exons. When *Ds1* provides both a donor and an acceptor, the exonized transcript combines the 1st to the *i* th exons, the first *j* nt of the *i* th intron, the *Ds1* sequences until the junction site, the 8 nt upstream sequence, the *i* th intron starting from the (*j* + 1) nt to the end, and the sequences of the (*i* + 1)th to the (*I* + 1)th exons. Note the 8 nt upstream sequence contains the last 8 nt of the joined sequence of the 1st to the ith exons and the first *j* nt of the ith *i* ntron.

### Analysis of exonized transcript variants and prediction of isoforms

All exonized transcripts were assigned for open reading frame (ORF) analysis starting at the original start codon and terminating at the first in-frame stop codon. The transcripts were designated type I, II, III, or IV, if the in-frame stop codon occurred at the conserved region in the original splice junction, the intron inserted by *Ds1*, the *Ds1*, or any exon after *Ds1* insertion, respectively. If no in-frame stop codon was found during ORF analysis, the corresponding transcript was designated type V, and the incomplete transcript without a stop codon was output directly. All transcripts containing a termination codon more than 55 nt upstream of the last exon/exon junction were considered putative targets for the NMD pathway (Chang et al., 2007; Hori and Watanabe, 2007) and were excluded from isoform prediction.

The proteins for transcripts not targeted to the NMD pathway were further classified into 2 subtypes: an interior isoform if the termination codon was the same as the reference transcript (the transcript without *Ds1* insertion); otherwise, a C-terminal isoform. For an interior variant, the number of additional peptides inserted in the middle was recorded. For a C-terminal variant, its similarity to the corresponding reference protein was defined by the fraction of the number of peptides in the 2 sequences being identical to the total length of the reference protein.

## Results

More than half of *Ds1* exonized transcripts undergo the NMD pathway or yield truncated protein isoforms without a TE genetic message.

Previous study had revealed that an exonic *Ds1* can provide splice donor as well as acceptor sites for alternative splicing (Wessler, 1991). A genome-wide computational analysis to simulate all possible *Ds1* exonized transcripts was performed accordingly in each intron of rice and Arabidopsis genes and yielded 422,960,068 and 196,284,528 exonized transcripts, respectively (Additional file 1: Table S1 and Table S2). The resulting transcripts in each genome were classified into 5 types by the locations of PTC (Figure 1).

**Figure 2:**
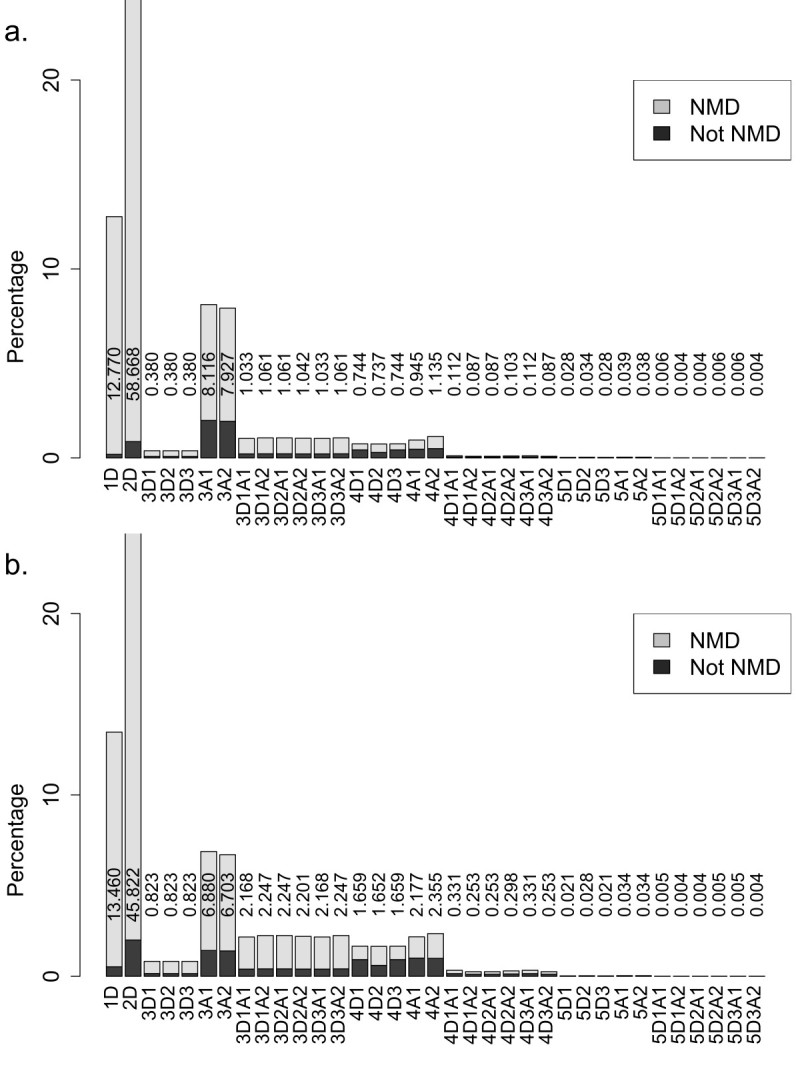
**Proportions of 5 types (named initiating with 1 to 5) of exonized transcripts for** ***Ds1*** **insertion on different chromosomes leading to the nonsense-mediated decay (NMD) or non-NMD pathway in a) rice and b) Arabidopsis.** The usage of *Ds1* donor and/or acceptor for exonization are presented following each type. Accordingly, type 1 and 2 transcripts consist of splice donors only.

Type I exonized transcripts of *Ds1* carry a PTC in frame in the splice donor consensus. This indicates that those exonized transcripts resulted from *Ds1* merely offering acceptors will not yield type I transcripts. In plants, a PTC in the splice donor consensus is represented as CAG/GTAAGT in plants (Baek et al., 2008; Schuler 2008). Therefore, about one-third of the exonized transcripts created by skipping this donor consensus carry a TAA termination codon in frame. Similarly, type II exonization events have in-frame PTCs within the *Ds1* inserting introns but outside the original donor consensus. Neither types I nor II transcripts yield truncated protein isoforms with any genetic message of *Ds1*. Genome-wide computational analysis revealed that, when the splice donor site upstream the *Ds1* is skipped, 12.8% and 58.7% of the exonized transcripts in rice are Type I and II, respectively (Additional file 1: Table S1 and Figure 2a). In Arabidopsis, 13.5% and 45.8% of exonization evens are type I and II, respectively (Additional file 1: Table S2 and Figure 2b).

### *Ds1* or subsequent flanking intron offers the termination codons of the exonized transcripts

The TE itself may contain PTCs upstream of the donor sites or downstream the acceptor sites. This results in the third type of exonization events, whereby the inserted *Ds1* offers the in-frame termination codon of the exonized transcripts. According to previous studies, *Ds1* provides 3 donors (maximal 35 nucleotides from the donors-end) and 2 acceptors (24 nucleotides from the acceptors-end for the subsequent transcripts). This yielded 11 transcript isoforms for each *Ds1* insertion. For type III transcripts in rice, when *Ds1* provides one splice site alone, i.e. either donor (3D1-3D3) or acceptor (3A1 and 3A2), 3D1-3D3 account for about 2.5% while 2As’ account for about 13.6%. About 23.5% of the total simulated transcripts are type III, including 18.0% that are targets of NMD (Figure 2). Even though the other 5.5% are non-NMD targets, they would yield the protein isoforms with up to 59 bp of *Ds1* messages. As shown in Figure 1a, *Ds1* begins with 2 discontinuous but in-framed PTCs. About 45.9% of the total type III transcripts were determined by the first PTC of *Ds1*. This kind of transcripts will yield truncated protein isoforms without any genetic message of *Ds1*. For the other 54.1% of type III transcripts, the PTCs locate on the inserted intron sequences downstream the *Ds1*.

### With type IV *Ds1* exonization, PTCs locate in exons downstream the *Ds1*

The above results as well as our previous studies indicated that the major potential protein isoforms caused by *Ds1* exonization depend on the location of termination codons downstream the *Ds1* transposon. Type IV involves events in which PTCs in the original transcripts become in-frame in the simulated transcripts. *Ds1* inserted in introns would yield 11 different patterns of type IV transcripts of a single *Ds1* insertion site. In order to clarify the effect of *Ds1* exonization in type IV transcripts, we divided the outcomes into 3 groups: group D providing donor alone, group A providing acceptor alone; and group DA providing a donor and an acceptor. For type IV transcripts, the outcomes were termed 4D1, 4D2, 4D3 (when using donors only for exonization); 4A1, 4A2 (when using acceptor only for exonization); and D1A1, D1A2, D2A1, D2A2, D3A1 and D3A2. Among all type IV transcripts, about 51.4% were non-NMD targets and would yield protein isoforms. The translated products of transcripts D1A1, D1A2, D2A1, D2A2, D3A1 and D3A2 will retain the genetic messages of the intron which *Ds1* inserted in. Type V exonization events indicate simulated transcripts harboring no in-frame termination codon until the end of the target gene. In rice, these occur with low frequencies: less than 0.2% (Figure 2). Exonized transcripts of Type V may enhance the functional protein isoforms of the *Ds1*-inserted genes.

### Characterization of protein isoforms created by *Ds1* exonized transcripts

**Figure 3:**
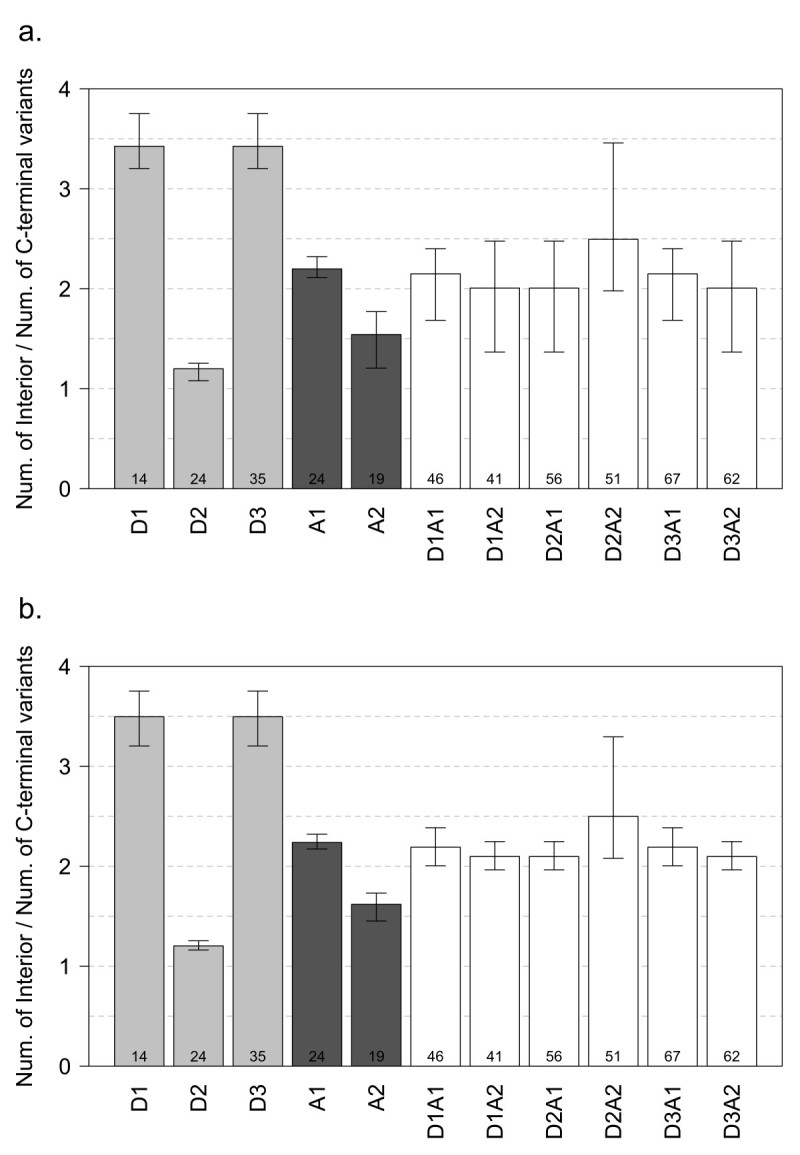
**Ratios of interior to C-terminal protein isoforms for type IV transcripts with** ***Ds1*** **insertion in a) rice and b) Arabidopsis.** Data are mean ratios over all chromosomes (whiskers are range). Numbers shown on the base of each column indicated the integrated *Ds1* message plus the duplicated target site (8 nucleotides). The remainder of each number divided by 3, i.e. 0, 1 or 2, determines the pattern of interior/C-terminal ratio in each *Ds1* providing type.

Although many simulated exonization events are predicted to produce different protein isoforms, their extent needs further analysis. According to our previous identification, the translated protein isoforms were characterized as C-terminal or interior variants. For C-terminal isoforms, peptides from the new reading frame replace the C terminus of the reference protein. For interior isoforms, the simulated transcripts have the same termination codon as the reference transcripts do. In these transcripts, the upcoming transcripts of the exonized junction have the same reading frame as the reference gene, and therefore, the translation products of the TE and intron transcripts act as a “peptide insertion” in the reference gene products. All type V transcripts yielded C-terminal isoforms. A type IV transcript, however, may produce a C-terminal or interior variant. Figure 3 shows the ratio of interior to C-terminal simulated isoforms of type IV non-NMD proteins in rice and Arabidopsis. Among 11 exonization patterns, the ratio of D1 is the same to D3, D1A1 to D3A1. Similarly, D1A2, D2A1 and D3A2 show the same ratio (see Discussion).

To analyze the homology to the reference proteins, C-terminal variants were further graded by the proportion of the amino acids in the reference sequences containing in the isoform, which presented as <25% (Very low), 25%-50% (Low), 50%-75% (Medium) and >75% (High). In general, the proportions of H and M variants were more than 40% and 20%, respectively.

**Figure 4:**
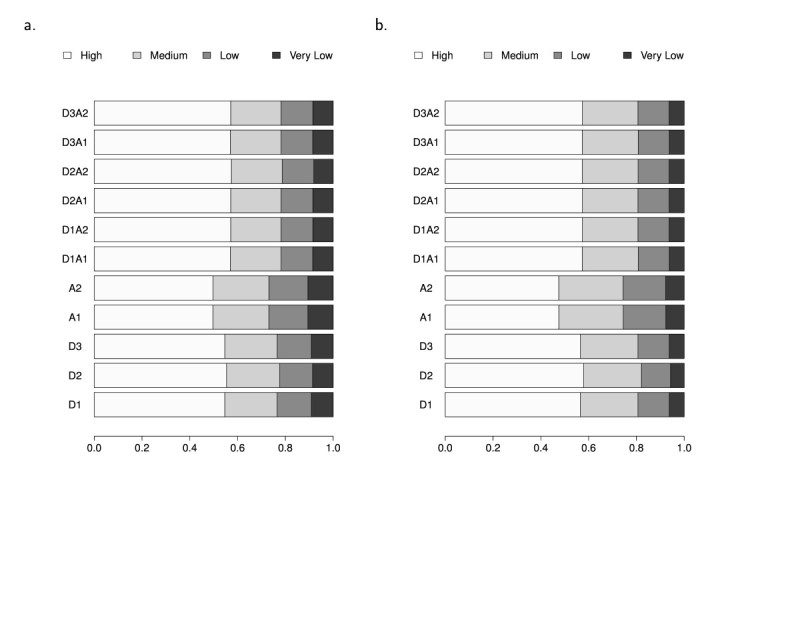
**Number of additional amino acids in the interior protein isoforms in a) rice chromosome 10 and b) Arabidopsis chromosome 4.** ‘Min’ and ‘max’ are the minimum and maximum number of additional amino acids, respectively; p15, p30, and p50 are the cumulative proportions of interior variants with additional number of amino acids ≤ 15, ≤ 30, and ≤ 50, respectively.

For the interior variants, Figure 4 showed the length of inserted peptides for all simulated interior isoforms from rice and Arabidopsis chromosomes. The number of amino acids of insertion can vary from 5 to 1137 (data not shown) while most interior isoforms contain short insertions.

## Discussion

Our previous studies suggested that the main contribution of *Ds* exonization to gene diversity is, based on a single transposition event, providing different splice donors for different reading frames rather than providing genetic messages (Huang et al., 2012). According to this, we performed a genome-wide computational approach to assess the role of *Ds* exonization in plants. We simulated a bulk of *Ds* exonized transcript orthologs from the dicot *Arabidopsis thaliana* and the monocot *Oryza sativa* (Liu and Charng, 2012). In addition, the protein isoforms are classified as C-terminal or interior variants to reveal the possible complexity of the proteome caused by *Ds* exonization. However, its extend is limited by the fact that *Ds* is biased towards providing donors for exonization. Blasting analyses indicate many other members of *Ac*/*Ds* family, e.g. *Ds2* (D1-6) transposons, contain the same splice consensuses. However, *Ds1* transposon shows a different pattern, which has been reported to offer 3 splice donors and 2 acceptors when it exists in an exon. Therefore, it is expected that *Ac*/*Ds* family can enrich the plant proteome with two forms, one by *Ds* members and the other by *Ds1* members. This aspect encourages us to reveal the effects of the exonization events which occurred by *Ds1*, i.e. either combined usage of one donor and one acceptor or single usage of one donor or one acceptor. In this report, genome-wide exonized transcript orthologs with *Ds1* insertion from rice and Arabidopsis were simulated to study their impact on proteome complexity in plants. Unlike *Ds*, whose both forward and reverse forms contain splice sites (Huang et al., 2012), all splice sites of *Ds1* were observed in reverse pattern. Therefore, all simulated transcripts were created by presuming that *Ds1* inserts in introns in the reverse pattern. According to our previous report, we set an equal probability of the *Ds1* exonization in each position to simulate all possible exonized transcripts for assessing the extent to which it leads to proteome expansion. These yielded 422,960,068 transcripts from rice and 196,284,528 from Arabidopsis for further analysis. Previously, we studied the exonization effect of a single inserted site by *Ds* providing 5 splice donor sites (1 for forward and 4 for reverse pattern), which may result in 5 exonized transcripts. Contrarily, *Ds1* provide 3 donors and 2 acceptors, which resulting 11 additional transcript isoforms by a single insertion event. Therefore, *Ds1* exonization in plants may yield more than 2-fold number of transcripts isoforms than *Ds* exonization does. Also, *Ds1* differs to *Ds* at position 20 leads to abortion of another PTC (Figure 1), which will greatly decrease the proportion of the NMD pathway of the exonized transcripts (see below). Thus, the effects of *Ds1* exonization harbor more extent than *Ds* exonization. To study whether part of *Ds1* sequence may be found out in the plant full length cDNA data, we performed a BLAST search of *Ds1* sequences by running megablast (BLASTN 2.2.27+) in the NCBI nr nucleotide database for vascular plants (txid58023). There were 63 matched transcripts resulting from the exonization events either by providing donor/acceptor alone or both (Additional file 1: Table S3).

**Figure 5:**
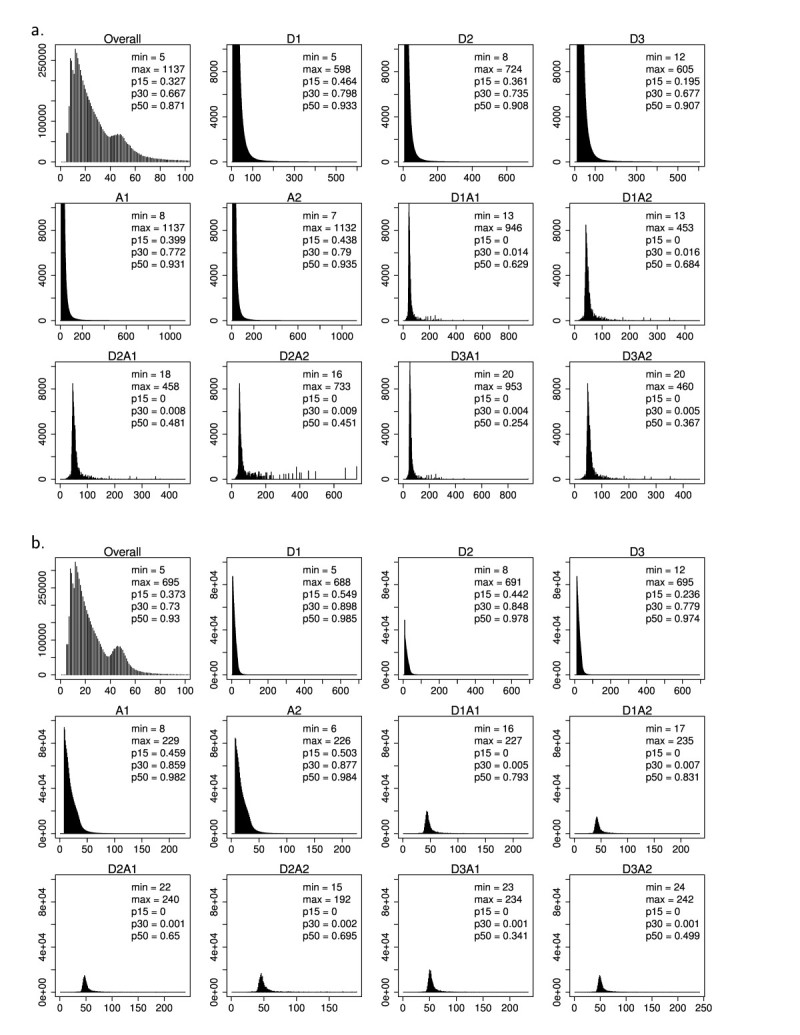
**Distribution of C-terminal variants among Type III, IV and V non-NMD transcripts in a) rice and b) Arabidopsis.** <25% (Very low), 25%-50% (Low), 50%-75% (Medium) and >75% (High).

To assess the proteome expansion of *Ds1* exonization, the first step is to exclude the exonized transcripts that contain a PTC, which can trigger the decay of the transcript through the NMD pathway. According to the location of termination codons, we classified the exonized transcripts into 5 types (Figure 2). Similar distributions had been shown in rice and Arabidopsis; only the results obtained in rice were discussed in this section. More than 70% of all exonized transcripts are types I and II that would yield no translation product or a truncated protein isoform without TE genetic message. The transcripts from types III to V were discussed respectively according to the splice donor and/or acceptor that the *Ds1* provides. Each *Ds1* insertion yielded 11 transcript isoforms. For type III transcripts, when *Ds1* provides one splice site alone, i.e. either donor (3D1-3D3) or acceptor (3A1 and 3A2), 3D1-3D3 account for about 1% while 2As’ for 16%. That results in about 20 times isoforms of a 3A*i* (*i* = 1, 2) versus a 3D*j* (*j* = 1, 2, 3), in average. It is because the three donor sites locate at 12, 22 or 33 bp from the *Ds1* terminus and *Ds1* begins with a stop codon (TAG). Contrarily, when *Ds1* provides acceptor but no donor for exonization, the resulting transcripts contain no intron sequences upstream the inserted *Ds1* and only a maximum of 24 bp of *Ds1* was transcribed without stop codon. The stop codons of 3A1 and 3A2 transcripts can only originated from the inserted intron sequences, which are downstream the *Ds1*. Types III and V exonized transcripts may carry a part of genetic information from TE. All type V transcripts and non-NMD type IV transcripts should be the main sources of new protein isoforms of the reference genes. We further characterized the translation products of these transcripts. Isoforms generated by exonization often contain an additional protein sequence due to a shift of the reading frame and/or unspliced intron-TE region. The translated protein isoforms were characterized as C-terminal or interior variants. As shown in Figure 2, the non-NMD transcripts of type III, especially 3A1 and 3A2, are the main source of C-terminal variants, which actually are truncated forms of the reference proteins. The number of the translated protein isoforms yielded by type IV and V non-NMD exonized transcripts, 10,881,168 (about 2.6% of the total), is much more than the one that *Ds* transposon yielded (Liu and Charng, 2012). From a total of 4,008,051 C-terminal variants, 59.1% and 17.2% showed high and medium similarity to their reference proteins, respectively (Figure 5). By retaining the (major) portion of the reference protein, these variants may provide modified peptides (at C-terminal) yet functional isoforms for selective advantage. Other 23.7% of the C-terminal variants, designated to have low (L) or very low (V) similarity with the reference protein, mostly result from *Ds1* insertions in the first few introns. These variants may retain the less functional domains of their reference proteins. However, these L and V variants may still act as functional proteins. For example, 2nd intronic Alu-exonized C-terminal variants code for functional isoforms, which were determined as new members of the reference protein (Liu et al., 2002; Wu et al., 2007; Yi et al., 2003). In order to survey the compositions of protein isoforms translated by the transcript variants, we arranged the results of the protein isoforms with 3 groups: group 1 providing donor alone, group 2 providing acceptor alone, and group 3 providing a donor and an acceptor. In Figure 3, group 3 is composed of 6 combinations but shown 3 distinct interior/C-terminal ratios: 3.459, 2.233 and 1.549. In fact, those combinations harboring the same remainder of the number of incorporated nucleotides (shown on the base of each column) divided by 3 would translate protein isoforms sharing the most amino acids sequences of the incorporated messages. The fact of the same remainder also contributes to the feature of 3 distinct patterns (D2A2, {D1A1, D3A1}, {D1A2, D2A1, D3A2}) of interior proteins from the 6 combinations (Figure 4). For a single *Ds1* insertion event, providing one donor and one acceptor indicates incorporating the inserted introns messages, both upstream and downstream the *Ds1* plus an additional duplicate insertion site sequences (8 nucleotides). Although 6 different transcript isoforms could be yielded, only a maximal 3 distinct protein isoforms would be translated. Each one of the protein isoforms yielded by D1A2, D2A1 and D3A2 differs with the others by 2, 5 or 7 amino acids. Whether these differences lead to independent protein variants to the reference protein needs further analysis, whose results would reveal whether *Ds1* exonization triggers more proteome expansion than *Ds* does.

## Conclusions

In conclusion, the main contribution of *Ds1* and *Ds* exonization for gene divergence is not providing genetic messages but incorporating the intron sequences with different reading frame patterns to enrich the plant proteome. All these simulation results direct new experimental analysis at the molecular level.

## Electronic supplementary material

Additional file 1: Table S1: Number of exonized transcripts for *Ds1* insertion on different chromosomes in rice genome. **Table S2.** Number of exonized transcripts for *Ds1* insertion on different chromosomes in Arabidopsis genome. **Table S3.** Identification of the plant cDNAs containing the exonization donor/acceptor sequences of *Ds1* transposable element. Sequence for donor (D1, D2, D3) and acceptor (A1 or A2) were blast, either alone or combined, with the cDNA data bases. Note that the D3 sequences cover the sequences of D1 or D2, and the A1 sequences cover the ones of A2. (PDF 111 KB)

Below are the links to the authors’ original submitted files for images.

Authors’ original file for figure 1

Authors’ original file for figure 2

Authors’ original file for figure 3

Authors’ original file for figure 4

Authors’ original file for figure 5

## Abbreviations

TE: Transposable element
AS: Alternative splicing
PTC: Premature termination codon
NMD: Nonsense-mediated decay.
