# Supplementary material for: The extent of Ds1 transposon to enrich transcriptomes and proteomes by exonization
Source: Bot Stud. 2013 Aug 21;54:14. doi: 10.1186/1999-3110-54-14 (PMC5432752; doi:10.1186/1999-3110-54-14)
Supplement: Supplementary file 1 — Additional file 1: Table S1: Number of exonized transcripts for Ds1 insertion on different chromosomes in rice genome. Table S2. Number of exonized transcripts for Ds1 insertion on different chromosomes in Arabidopsis genome. Table S3. Identification of the plant cDNAs containing the exonization donor/acceptor sequences of Ds1 transposable element. Sequence for donor (D1, D2, D3) and acceptor (A1 or A2) were blast, either alone or combined, with the cDNA data bases. Note that the D3 sequences cover the sequences of D1 or D2, and the A1 sequences cover the ones of A2. (PDF 111 KB) [file 40529_2012_17_MOESM1_ESM.pdf]

Table S1. Number of exonized transcripts for *Ds1* insertion on different chromosomes in rice genome.

	Chromosome 1		Chromosome 2		Chromosome 3		Chromosome 4		Chromosome 5		Chromosome 6		Chromosome 7		Chromosome 8		Chromosome 9		Chromosome 10		Chromosome 11		Chromosome 12		
	nonNMD	NMD	nonNMD	NMD	nonNMD	NMD	nonNMD	NMD	nonNMD	NMD	nonNMD	NMD	nonNMD	NMD	nonNMD	NMD	nonNMD	NMD	nonNMD	NMD	nonNMD	NMD	nonNMD	NMD	Total
1D1	52014	2463795	43680	2052489	45156	2245116	31734	1513173	28137	1361739	28266	1435347	24864	1441365	22866	1127727	21741	1014267	17187	866361	19344	1081035	20052	1046955	18004410
1D2	39579	2476230	33108	2063061	34260	2256012	24189	1520718	21447	1368429	21420	1442193	18906	1447323	17397	1133196	16485	1019523	13068	870480	14682	1085697	15261	1051746	18004410
1D3	25959	2489850	21747	2074422	22437	2267835	15906	1529001	14058	1375818	13992	1449621	12405	1453824	11409	1139184	10788	1025220	8541	875007	9582	1090797	9981	1057026	18004410
2D1	223020	11068332	186972	9460644	206184	10223739	139449	6843426	130134	6372717	126237	6528393	125973	6140757	112821	6032916	88395	4861125	82725	4120149	88407	4786458	89130	4676394	82714497
2D2	172257	11119095	144891	9502725	159489	10270434	107859	6875016	100449	6402402	97407	6557223	97326	6169404	87798	6057939	68376	4881144	63852	4139022	68145	4806720	68817	4696707	82714497
2D3	114696	11176656	96480	9551136	106125	10323798	71685	6911190	66738	6436113	64695	6589935	64866	6201864	58722	6087015	45549	4903971	42366	4160508	45156	4829709	45777	4719747	82714497
3D1	46915	183359	40538	161047	41821	171880	29252	109019	29230	105699	27763	98897	27538	95886	22843	86990	17809	67227	18211	61116	18240	62865	18240	62865	1605250
3D2	46246	184028	40237	161348	41158	172543	28996	109275	28797	106132	27440	99220	26985	96439	22480	87353	17590	67446	17860	61467	18047	63058	18047	63058	1605250
3D3	45645	184629	40020	161565	40931	172770	28723	109548	28662	106267	27329	99331	26618	96806	22437	87396	17443	67593	17698	61629	17991	63114	17991	63114	1605250
3A1	1078480	3627032	970894	3029520	1022562	3301085	720406	2175078	657226	2039856	671323	2084592	626824	2008093	612774	1875929	543017	1491172	475687	1259053	508221	1520584	508221	1520584	34328213
3A2	1055280	3532194	954633	2972034	987827	3222823	709184	2137960	648059	1997428	655628	2019531	614936	1952711	587261	1842299	529329	1461867	468477	1228743	494998	1479302	494998	1479302	33526804
3D1A1	123229	501212	107902	439690	110833	466002	77213	298131	77979	289138	74578	270605	71909	262465	61095	239428	47752	186208	49186	168422	49920	174128	49920	174128	4371073
3D1A2	127369	515521	111593	451532	113324	480514	80384	305386	80139	295624	76849	276885	74230	270183	62528	244927	49183	189141	50035	172885	50418	178255	50418	178255	4485578
3D2A1	127367	515523	111593	451532	113324	480514	80384	305386	80138	295625	76849	276885	74227	270186	62528	244927	49183	189141	50035	172885	50418	178255	50418	178255	4485578
3D2A2	123586	507778	108439	443066	111338	472711	79075	298587	77557	294308	73887	273629	71460	265599	62106	241706	48693	185968	48707	169921	49413	174781	49413	174781	4406509
3D3A1	123227	501214	107901	439691	110833	466002	77213	298131	77978	289139	74578	270605	71908	262466	61095	239428	47752	186208	49186	168422	49920	174128	49920	174128	4371073
3D3A2	127367	515523	111592	451533	113324	480514	80384	305386	80138	295625	76849	276885	74227	270186	62528	244927	49183	189141	50035	172885	50418	178255	50418	178255	4485578
4D1	259742	193967	222246	172284	238355	181601	156018	114969	153748	110980	142164	105580	139783	101488	123122	92069	95198	72223	89983	64962	89821	68605	89821	68605	3147334
4D2	175737	274476	149354	242781	157225	257604	107699	160811	104651	157272	98768	146690	97323	141368	84123	129086	66169	99580	61313	91877	62836	94549	62836	94549	3118677
4D3	259742	193967	222246	172284	238355	181601	156018	114969	153748	110980	142164	105580	139783	101488	123122	92069	95198	72223	89983	64962	89821	68605	89821	68605	3147334
4A1	279659	293964	242239	262429	260612	281729	175679	176030	160568	169750	158174	163259	150335	153355	130710	134738	103461	107678	95186	97507	98909	101758	98909	101758	3998396
4A2	292622	399289	249712	328815	287616	368210	179533	220630	164441	217434	169009	233415	156922	214490	151535	173369	112364	141485	99538	130791	110182	145043	110182	145043	4801670
4D1A1	33935	37318	29499	31045	32517	35318	19806	21766	19144	21151	18306	19159	18218	19239	14397	16550	10643	12351	10114	11377	10281	10950	10281	10950	474315
4D1A2	26467	27348	21356	24934	22631	28483	15502	16690	14594	17675	14059	15362	13460	15164	12235	12464	9084	9913	8235	8429	7905	8614	7905	8614	367123
4D2A1	26467	27348	21356	24934	22631	28483	15502	16690	14594	17675	14059	15362	13460	15164	12235	12464	9084	9913	8235	8429	7905	8614	7905	8614	367123
4D2A2	30291	32714	26687	30093	27693	32865	21784	18173	17809	17910	17070	17477	17505	17515	12883	15356	9790	12174	10518	9814	10796	9618	10796	9618	436949
4D3A1	33935	37318	29499	31045	32517	35318	19806	21766	19144	21151	18306	19159	18218	19239	14397	16550	10643	12351	10114	11377	10281	10950	10281	10950	474315
4D3A2	26467	27348	21356	24934	22631	28483	15502	16690	14594	17675	14059	15362	13460	15164	12235	12464	9084	9913	8235	8429	7905	8614	7905	8614	367123
5D1	14609	0	15206	0	14204	0	10323	0	9507	0	10014	0	9714	0	8116	0	5641	0	6201	0	6589	0	6589	0	116713
5D2	18105	0	17601	0	19331	0	12800	0	12312	0	12300	0	12294	0	10098	0	7313	0	7956	0	7630	0	7630	0	145370
5D3	14609	0	15206	0	14204	0	10323	0	9507	0	10014	0	9714	0	8116	0	5641	0	6201	0	6589	0	6589	0	116713
5A1	21844	0	20834	0	21938	0	14982	0	12673	0	13151	0	13455	0	11099	0	7946	0	8514	0	8396	0	8396	0	163228
5A2	21594	0	20722	0	21450	0	14868	0	12711	0	12916	0	13003	0	10786	0	8229	0	8398	0	8343	0	8343	0	161363
5D1A1	2898	0	3185	0	3191	0	2665	0	1752	0	1770	0	2578	0	1670	0	1144	0	1374	0	841	0	841	0	23909
5D1A2	1887	0	1906	0	2909	0	1619	0	1132	0	1263	0	1372	0	986	0	777	0	889	0	928	0	928	0	16596
5D2A1	1887	0	1906	0	2909	0	1619	0	1132	0	1263	0	1372	0	986	0	777	0	889	0	928	0	928	0	16596
5D2A2	4223	0	3036	0	3254	0	1962	0	1580	0	2355	0	2330	0	1089	0	1473	0	1513	0	1512	0	1512	0	25839
5D3A1	2898	0	3185	0	3191	0	2665	0	1752	0	1770	0	2578	0	1670	0	1144	0	1374	0	841	0	841	0	23909
5D3A2	1887	0	1906	0	2909	0	1619	0	1132	0	1263	0	1372	0	986	0	777	0	889	0	928	0	928	0	16596
Total	5203741	53107028	4572463	45212613	4833199	48933987	3340330	32543595	3129091	30311712	3089307	30906182	2953451	29519231	2697284	27720466	2239848	22546166	2058508	19236909	2153487	22463061	2157189	22031220	422960068

Table S2. Number of exonized transcripts for *Ds1* insertion on different chromosomes in Arabidopsis genome.

	Chromosome 1		Chromosome 2		Chromosome 3		Chromosome 4		Chromosome 5		
	nonNMD	NMD	nonNMD	NMD	nonNMD	NMD	nonNMD	NMD	nonNMD	NMD	Total
1D1	120780	2294520	68418	1203123	86478	1528986	70962	1299012	109608	2024709	8806596
1D2	91890	2323410	51990	1219551	65808	1549656	53982	1315992	83313	2051004	8806596
1D3	60342	2354958	34080	1237461	43155	1572309	35358	1334616	54621	2079696	8806596
2D1	457770	7644240	247977	4262991	333321	5428794	261096	4408806	398856	6536460	29980311
2D2	358746	7743264	194472	4316496	261735	5500380	205503	4464399	312552	6622764	29980311
2D3	240876	7861134	130584	4380384	176556	5585559	138495	4531407	209994	6725322	29980311
3D1	78842	360497	44443	187605	59733	257195	46193	199248	69656	312985	1616397
3D2	77915	361424	43889	188159	59070	257858	45496	199945	68974	313667	1616397
3D3	77361	361978	43742	188306	58769	258159	45226	200215	68604	314037	1616397
3A1	732711	2930456	431607	1580526	523486	2046942	436364	1658418	668042	2496206	13504758
3A2	717991	2838803	424880	1539536	515330	1992928	428933	1613161	660483	2424235	13156280
3D1A1	207356	949266	116007	495312	155486	678787	119530	524669	183496	825362	4255271
3D1A2	212816	985847	120266	514674	160209	703714	124274	543478	188824	856682	4410784
3D2A1	212816	985847	120266	514674	160209	703714	124272	543480	188824	856682	4410784
3D2A2	207904	964518	115854	506750	156788	690164	120330	533097	185852	838285	4319542
3D3A1	207356	949266	116007	495312	155486	678787	119529	524670	183496	825362	4255271
3D3A2	212816	985847	120266	514674	160209	703714	124272	543480	188824	856682	4410784
4D1	487966	396513	260389	207606	354701	282513	276929	218463	427091	344361	3256532
4D2	317582	564191	172500	292912	233565	400701	183261	310013	275287	492926	3242938
4D3	487966	396513	260389	207606	354701	282513	276914	218478	427081	344371	3256532
4A1	532358	628189	281882	329155	387555	450789	304048	351496	462450	544990	4272912
4A2	527034	739795	279187	380213	382182	518517	299058	408918	452871	633855	4621630
4D1A1	78074	98423	41659	51852	57385	68989	46433	55129	68428	84034	650406
4D1A2	59290	75763	31651	38772	44072	53464	33585	44490	51024	64570	496681
4D2A1	59290	75763	31651	38772	44072	53464	33578	44497	51022	64572	496681
4D2A2	69512	90733	36314	45566	50385	63553	41718	50252	57075	79980	585088
4D3A1	78074	98423	41659	51852	57385	68989	46426	55136	68426	84036	650406
4D3A2	59290	75763	31651	38772	44072	53464	33578	44497	51022	64572	496681
5D1	11834	0	6391	0	8822	0	6253	0	8850	0	42150
5D2	14540	0	8974	0	11770	0	8371	0	12089	0	55744
5D3	11834	0	6391	0	8822	0	6253	0	8850	0	42150
5A1	17708	0	10767	0	13385	0	10052	0	14466	0	66378
5A2	17799	0	10121	0	13200	0	10308	0	14710	0	66138
5D1A1	2533	0	1604	0	2317	0	1325	0	1623	0	9402
5D1A2	1936	0	1071	0	1505	0	1259	0	1843	0	7614
5D2A1	1936	0	1071	0	1505	0	1259	0	1843	0	7614
5D2A2	2985	0	1950	0	2074	0	1689	0	1751	0	10449
5D3A1	2533	0	1604	0	2317	0	1325	0	1623	0	9402
5D3A2	1936	0	1071	0	1505	0	1259	0	1843	0	7614
Total	7120298	46135344	3944695	25028612	5209125	32434602	4124696	26239462	6285287	39762407	196284528

Table S3. Identification of the plant cDNAs containing the exonization donor/acceptor sequences of Ds1 transposable element. Sequences of donor (D1, D2 or D3) and acceptor (A1 or A2) were blast, either alone or combined, with the cDNA data bases. Note that the D3 sequences cover the sequences of D1 and D2, and the A1 sequences cover the ones of A2.

Query id	Subject ids	Subject Description	% identity	Alignment length	Mismatches	Gap opens	q. start	q. end	s. start	s. end	evalue	bit score
D3	gi|391416786|gb|BT085018.2|	Zea mays full-length cDNA clone ZM_BFb0227J12 mRNA, complete cds	100	35	0	0	1	35	108	74	7.00E-10	65.8
D3	gi|293337022|ref|NM_001176289.1|;gi|224031518|gb|BT067938.1|	Zea mays uncharacterized LOC100383644 (LOC100383644), mRNA >gb|BT067938.1| Zea mays full-length cDNA clone ZM_BFb0003C10 mRNA, complete cds	100	35	0	0	1	35	1361	1395	7.00E-10	65.8
D3	gi|224030094|gb|BT067226.1|	Zea mays full-length cDNA clone ZM_BFb0332P17 mRNA, complete cds	100	35	0	0	1	35	1190	1224	7.00E-10	65.8
D3	gi|223947208|gb|BT062991.1|	Zea mays full-length cDNA clone ZM_BFc0014G07 mRNA, complete cds	100	35	0	0	1	35	483	517	7.00E-10	65.8
D3	gi|226531467|ref|NM_001152610.1|;gi|219885596|gb|BT054566.1|	Zea mays putative O-Glycosyl hydrolase superfamily protein isoform 1 (LOC100279614), mRNA >gb|BT054566.1| Zea mays full-length cDNA clone ZM_BFc0158N11 mRNA, complete cds	100	35	0	0	1	35	492	526	7.00E-10	65.8
D3	gi|33321038|gb|AF488416.1|	Zea mays chromosome 9 BAC 9C20 complete sequence	100	35	0	0	1	35	17395	17361	7.00E-10	65.8
D3	gi|4102198|gb|AF010445.1|	Zea mays transposon Ds1 sequence	100	35	0	0	1	35	396	362	7.00E-10	65.8
D3	gi|55741046|gb|AY664414.1|	Zea mays cultivar B73 locus 9008, complete sequence	100	35	0	0	1	35	74095	74061	7.00E-10	65.8
D3	gi|22577|emb|X54710.1|	Z.luxurians DNA for DS-1 transposable element	100	35	0	0	1	35	9	43	7.00E-10	65.8
D3	gi|22477|emb|X59774.1|	Z.mays ruq-st transposon	100	35	0	0	1	35	396	362	7.00E-10	65.8
D3	gi|22476|emb|X59776.1|	Z.mays ruq66 transposon	100	35	0	0	1	35	1	35	7.00E-10	65.8
D3	gi|22260|emb|X07150.1|	Maize DNA for Ds101 controlling element (Ds1-related)	100	35	0	0	1	35	1	35	7.00E-10	65.8
D3	gi|22258|emb|X07148.1|	Maize DNA for Ds103 controlling element (Ds1-related)	100	35	0	0	1	35	1	35	7.00E-10	65.8
D3	gi|21924|emb|X07153.1|	Tripsacum dactyloides DNA for Ds132 element (Ds1-related)	100	35	0	0	1	35	1	35	7.00E-10	65.8
D3	gi|195655310|gb|EU975005.1|	Zea mays clone 462718 mRNA sequence	100	34	0	0	2	35	635	668	3.00E-09	63.9
D3	gi|22264|emb|X07154.1|	Maize DNA for Ds(bz-wm) controlling element (Ds1-related)	100	34	0	0	2	35	2	35	3.00E-09	63.9
D3	gi|22257|emb|X07147.1|	Maize DNA for Ds101 controlling element (Ds1-related)	100	34	0	0	2	35	1	34	3.00E-09	63.9
D3	gi|22211|emb|X14155.1|	Maize bronze bz1 gene 5' region with DS1 element Bz-wm inserted	100	34	0	0	2	35	26	59	3.00E-09	63.9
D3	gi|195593354|gb|EU941000.1|	Zea mays clone 1272747 mRNA sequence	100	32	0	0	4	35	850	881	3.00E-08	60.2
D3A1	gi|391416786|gb|BT085018.2|	Zea mays full-length cDNA clone ZM_BFb0227J12	100	35	0	0	1	35	108	74	7.00E-10	65.8
D3A1	gi|293337022|ref|NM_001176289.1|;gi|224031518|g	Zea mays uncharacterized LOC100383644	100	35	0	0	1	35	1361	1395	7.00E-10	65.8
D3A1	gi|224030094|gb|BT067226.1|	Zea mays full-length cDNA clone ZM_BFb0332P17 mRNA, complete cds	100	35	0	0	1	35	1190	1224	7.00E-10	65.8

Query id	Subject ids	Subject Description	% identity	Alignment length	Mismatches	Gap opens	q. start	q. end	s. start	s. end	evalue	bit score
D3A1	gi|223947208|gb|BT062991.1|	Zea mays full-length cDNA clone ZM_BFc0014G07 mRNA, complete cds	100	35	0	0	1	35	483	517	7.00E-10	65.8
D3A1	gi|226531467|ref|NM_001152610.1|;gi|219885596|gb|BT054566.1|	Zea mays putative O-Glycosyl hydrolase superfamily protein isoform 1 (LOC100279614),	100	35	0	0	1	35	492	526	7.00E-10	65.8
D3A1	gi|33321038|gb|AF488416.1|	Zea mays chromosome 9 BAC 9C20 complete sequence	100	35	0	0	1	35	17395	17361	7.00E-10	65.8
D3A1	gi|4102198|gb|AF010445.1|	Zea mays transposon Ds1 sequence	100	35	0	0	1	35	396	362	7.00E-10	65.8
D3A1	gi|55741046|gb|AY664414.1|	Zea mays cultivar B73 locus 9008, complete sequence	100	35	0	0	1	35	74095	74061	7.00E-10	65.8
D3A1	gi|22577|emb|X54710.1|	Z.luxurians DNA for DS-1 transposable element	100	35	0	0	1	35	9	43	7.00E-10	65.8
D3A1	gi|22477|emb|X59774.1|	Z.mays ruq-st transposon	100	35	0	0	1	35	396	362	7.00E-10	65.8
D3A1	gi|22476|emb|X59776.1|	Z.mays ruq66 transposon	100	35	0	0	1	35	1	35	7.00E-10	65.8
D3A1	gi|22260|emb|X07150.1|	Maize DNA for Ds101 controlling element (Ds1-	100	35	0	0	1	35	1	35	7.00E-10	65.8
D3A1	gi|22258|emb|X07148.1|	Maize DNA for Ds103 controlling element (Ds1-	100	35	0	0	1	35	1	35	7.00E-10	65.8
D3A1	gi|21924|emb|X07153.1|	Tripsacum dactyloides DNA for Ds132 element (Ds1-related)	100	35	0	0	1	35	1	35	7.00E-10	65.8
D3A1	gi|195655310|gb|EU975005.1|	Zea mays clone 462718 mRNA sequence	100	34	0	0	2	35	635	668	3.00E-09	63.9
D3A1	gi|22264|emb|X07154.1|	Maize DNA for Ds(bz-wm) controlling element (Ds1-related)	100	34	0	0	2	35	2	35	3.00E-09	63.9
D3A1	gi|22257|emb|X07147.1|	Maize DNA for Ds101 controlling element (Ds1-	100	34	0	0	2	35	1	34	3.00E-09	63.9
D3A1	gi|22211|emb|X14155.1|	Maize bronze bz1 gene 5' region with DS1 element Bz-wm inserted	100	34	0	0	2	35	26	59	3.00E-09	63.9
D3A1	gi|195593354|gb|EU941000.1|	Zea mays clone 1272747 mRNA sequence	100	32	0	0	4	35	850	881	3.00E-08	60.2
D3A2	gi|195655310|gb|EU975005.1|	Zea mays clone 462718 mRNA sequence	100	34	0	0	2	35	635	668	3.00E-09	63.9
D3	gi|386650819|gb|JQ887917.1|	Zea mays isolate Zheng58 clone 2 GRMZM2G701994-like gene, partial sequence	97.14	35	1	0	1	35	76	42	3.00E-08	60.2
D3	gi|22475|emb|X59775.1|	Z.mays ruq31 transposon	97.14	35	0	1	1	35	405	372	1.00E-07	58.4
D3A1	gi|386650819|gb|JQ887917.1|	Zea mays isolate Zheng58 clone 2	97.14	35	1	0	1	35	76	42	3.00E-08	60.2
D3A1	gi|22475|emb|X59775.1|	Z.mays ruq31 transposon	97.14	35	0	1	1	35	405	372	1.00E-07	58.4
D3A2	gi|386650819|gb|JQ887917.1|	Zea mays isolate Zheng58 clone 2 GRMZM2G701994-like gene, partial sequence	97.14	35	1	0	1	35	76	42	3.00E-08	60.2
D3A2	gi|22257|emb|X07147.1|	Maize DNA for Ds101 controlling element (Ds1-related)	95.56	45	2	0	2	46	1	45	4.00E-12	73.1

Query id	Subject ids	Subject Description	% identity	Alignment length	Mismatches	Gap opens	q. start	q. end	s. start	s. end	evalue	bit score
D3A2	gi|391416786|gb|BT085018.2|	Zea mays full-length cDNA clone ZM_BFb0227J12 mRNA, complete cds	93.48	46	3	0	1	46	108	63	6.00E-11	69.4
D3A2	gi|293337022|ref|NM_001176289.1|;gi|224031518|gb|BT067938.1|	Zea mays uncharacterized LOC100383644 (LOC100383644), mRNA >gb|BT067938.1| Zea	93.48	46	3	0	1	46	1361	1406	6.00E-11	69.4
D3A2	gi|224030094|gb|BT067226.1|	Zea mays full-length cDNA clone ZM_BFb0332P17 mRNA, complete cds	93.48	46	3	0	1	46	1190	1235	6.00E-11	69.4
D3A2	gi|223947208|gb|BT062991.1|	Zea mays full-length cDNA clone ZM_BFc0014G07 mRNA, complete cds	93.48	46	3	0	1	46	483	528	6.00E-11	69.4
D3A2	gi|226531467|ref|NM_001152610.1|;gi|219885596|g	Zea mays putative O-Glycosyl hydrolase	93.48	46	3	0	1	46	492	537	6.00E-11	69.4
D3A2	gi|33321038|gb|AF488416.1|	Zea mays chromosome 9 BAC 9C20 complete sequence	93.48	46	3	0	1	46	17395	17350	6.00E-11	69.4
D3A2	gi|4102198|gb|AF010445.1|	Zea mays transposon Ds1 sequence	93.48	46	3	0	1	46	396	351	6.00E-11	69.4
D3A2	gi|55741046|gb|AY664414.1|	Zea mays cultivar B73 locus 9008, complete	93.48	46	3	0	1	46	74095	74050	6.00E-11	69.4
D3A2	gi|22577|emb|X54710.1|	Z.luxurians DNA for DS-1 transposable element	93.48	46	3	0	1	46	9	54	6.00E-11	69.4
D3A2	gi|22477|emb|X59774.1|	Z.mays ruq-st transposon	93.48	46	3	0	1	46	396	351	6.00E-11	69.4
D3A2	gi|22476|emb|X59776.1|	Z.mays ruq66 transposon	93.48	46	3	0	1	46	1	46	6.00E-11	69.4
D3A2	gi|22260|emb|X07150.1|	Maize DNA for Ds101 controlling element (Ds1-related)	93.48	46	3	0	1	46	1	46	6.00E-11	69.4
D3A2	gi|22258|emb|X07148.1|	Maize DNA for Ds103 controlling element (Ds1-related)	93.48	46	3	0	1	46	1	46	6.00E-11	69.4
D3A2	gi|21924|emb|X07153.1|	Tripsacum dactyloides DNA for Ds132 element (Ds1-related)	93.48	46	3	0	1	46	1	46	6.00E-11	69.4
D3A2	gi|22264|emb|X07154.1|	Maize DNA for Ds(bz-wm) controlling element	93.33	45	3	0	2	46	2	46	2.00E-10	67.6
D3A2	gi|22211|emb|X14155.1|	Maize bronze bz1 gene 5' region with DS1 element	93.33	45	3	0	2	46	26	70	2.00E-10	67.6
D3A2	gi|195593354|gb|EU941000.1|	Zea mays clone 1272747 mRNA sequence	93.02	43	3	0	4	46	850	892	3.00E-09	63.9
D3A2	gi|22475|emb|X59775.1|	Z.mays ruq31 transposon	91.3	46	3	1	1	46	405	361	1.00E-08	62.1
