# Supplementary figures and images for: The extent of Ds1 transposon to enrich transcriptomes and proteomes by exonization
Source: Bot Stud. 2013 Aug 21;54:14. doi: 10.1186/1999-3110-54-14 (PMC5432752; doi:10.1186/1999-3110-54-14)

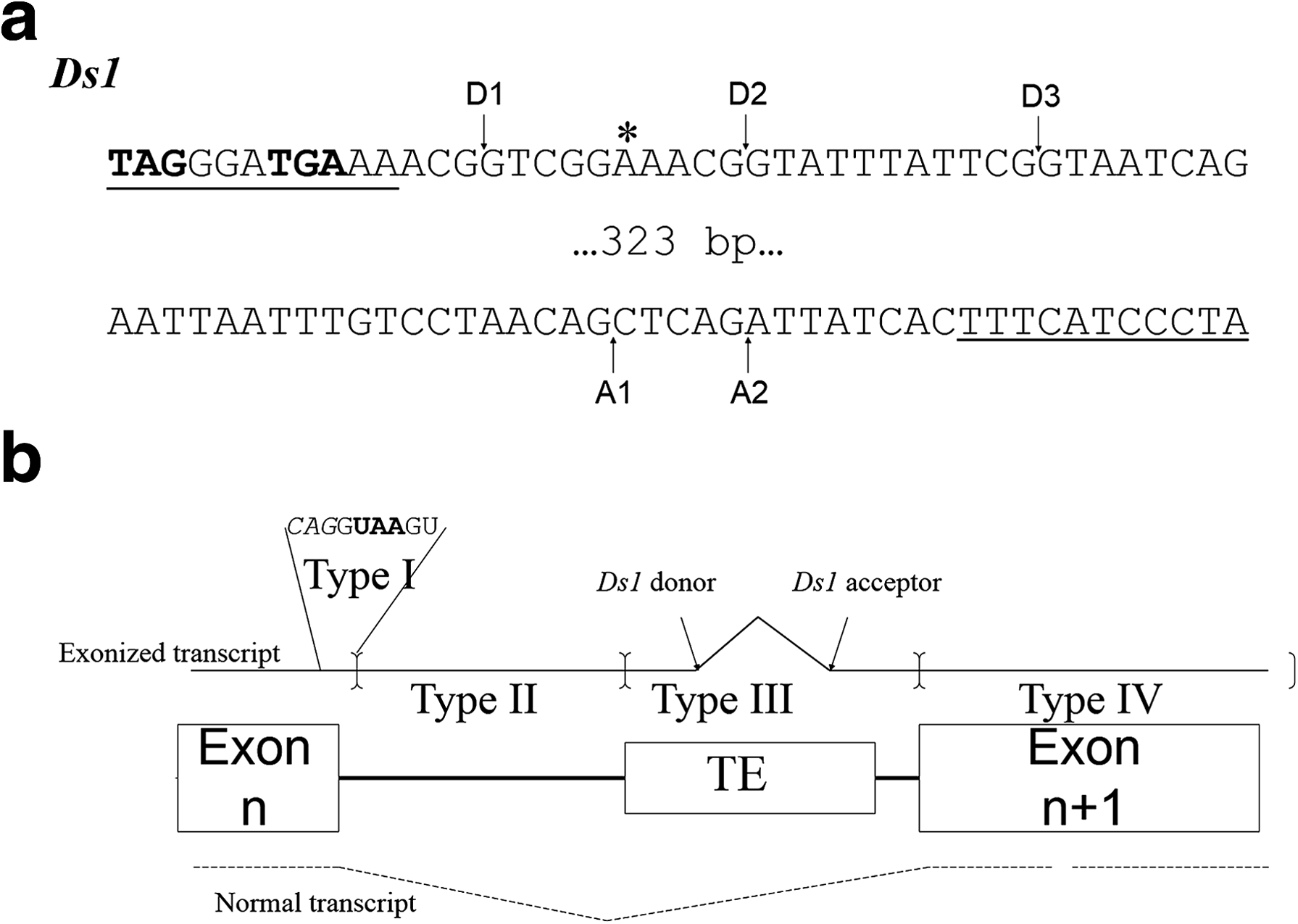

Supplement: Supplementary file 2 — Authors’ original file for figure 1 [file 40529_2012_17_MOESM2_ESM.tif]

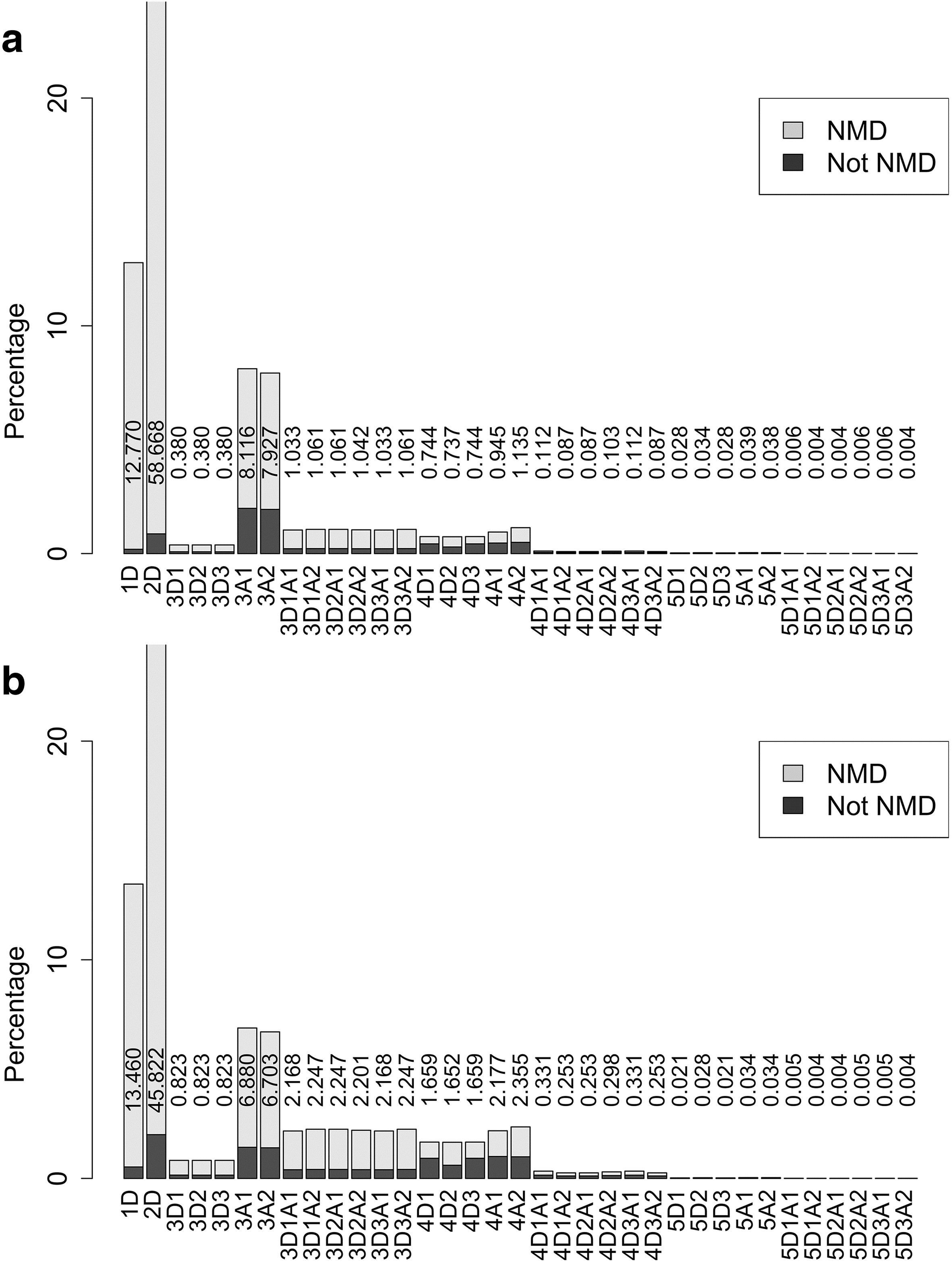

Supplement: Supplementary file 3 — Authors’ original file for figure 2 [file 40529_2012_17_MOESM3_ESM.tif]

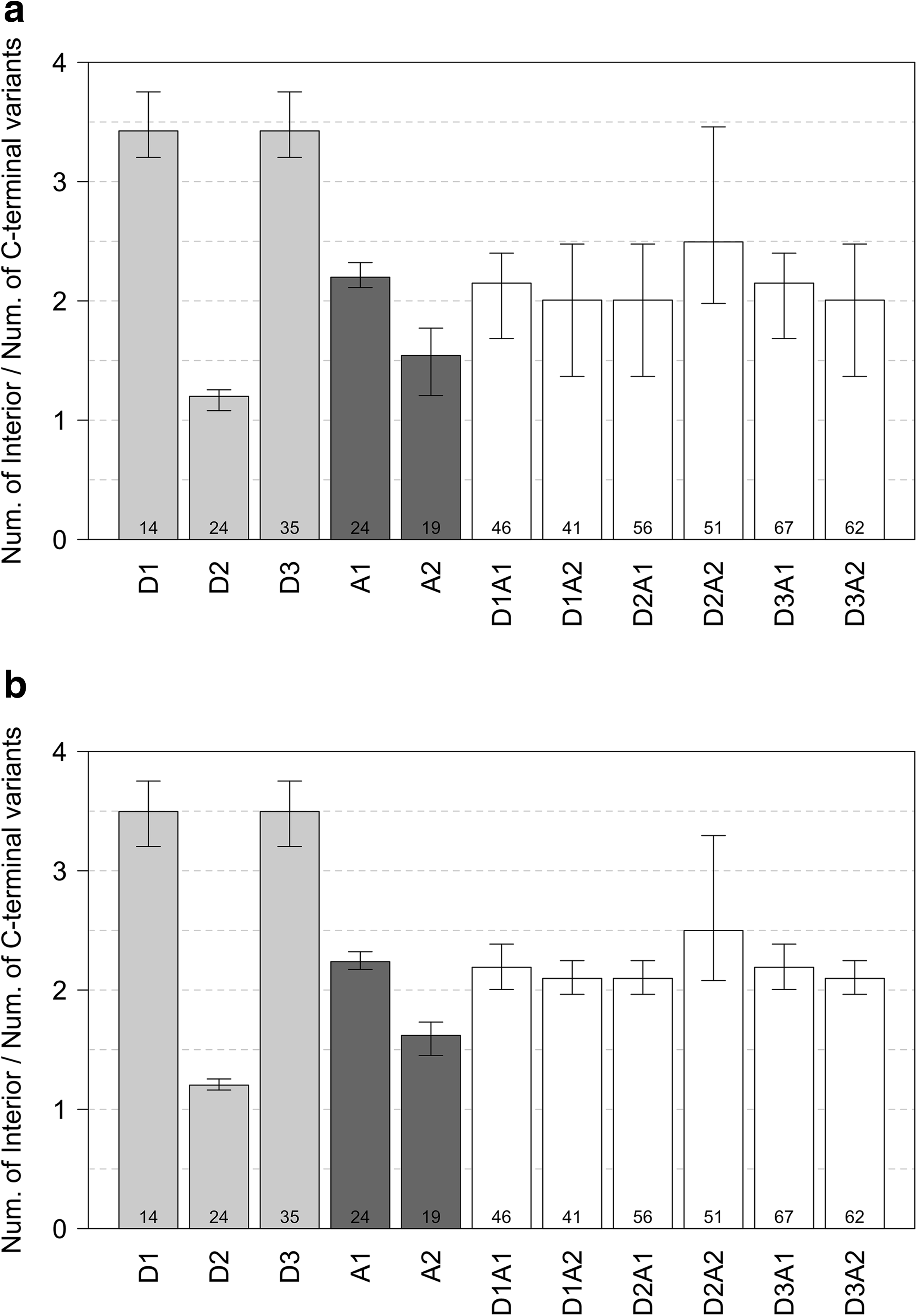

Supplement: Supplementary file 4 — Authors’ original file for figure 3 [file 40529_2012_17_MOESM4_ESM.tif]

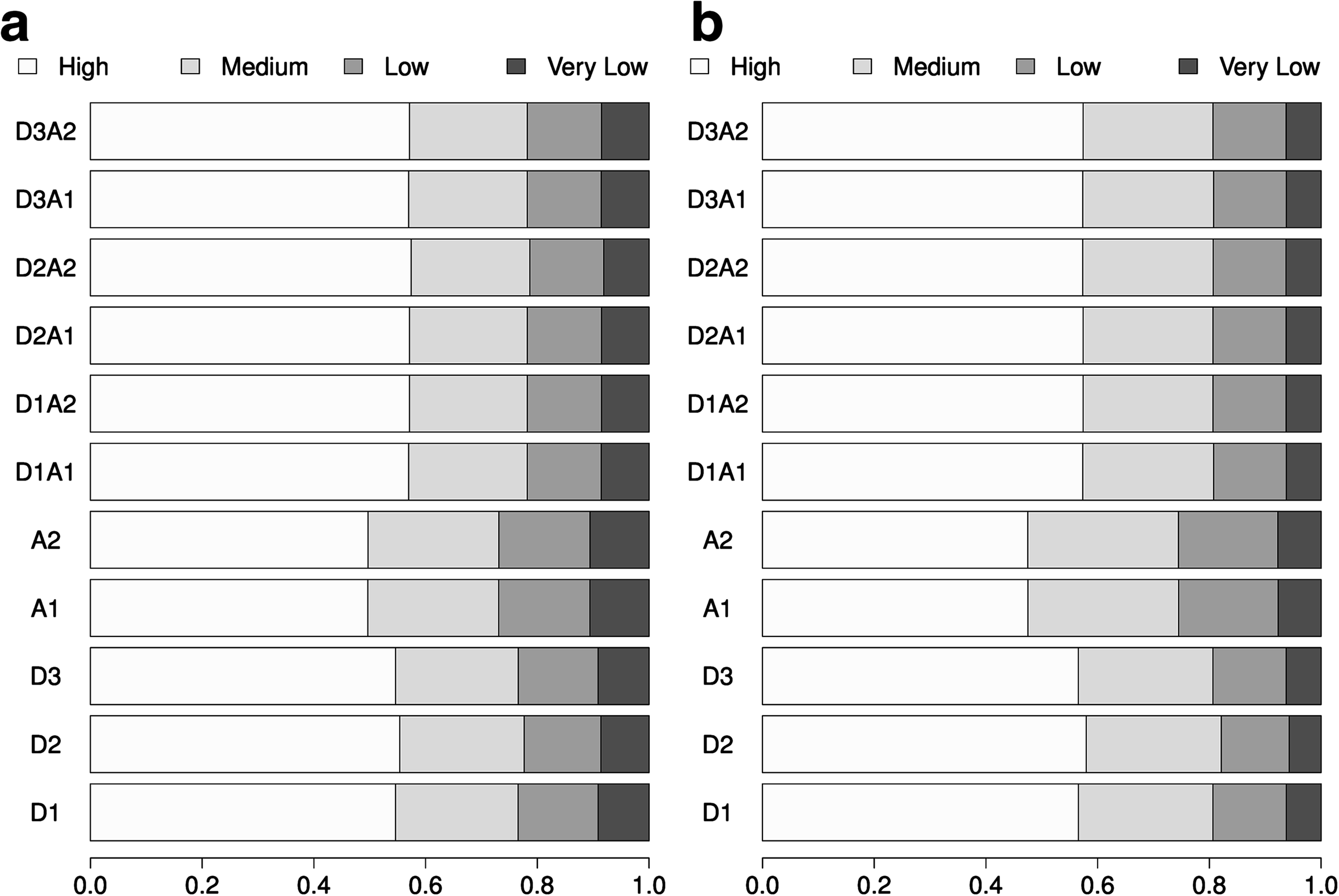

Supplement: Supplementary file 5 — Authors’ original file for figure 4 [file 40529_2012_17_MOESM5_ESM.tif]

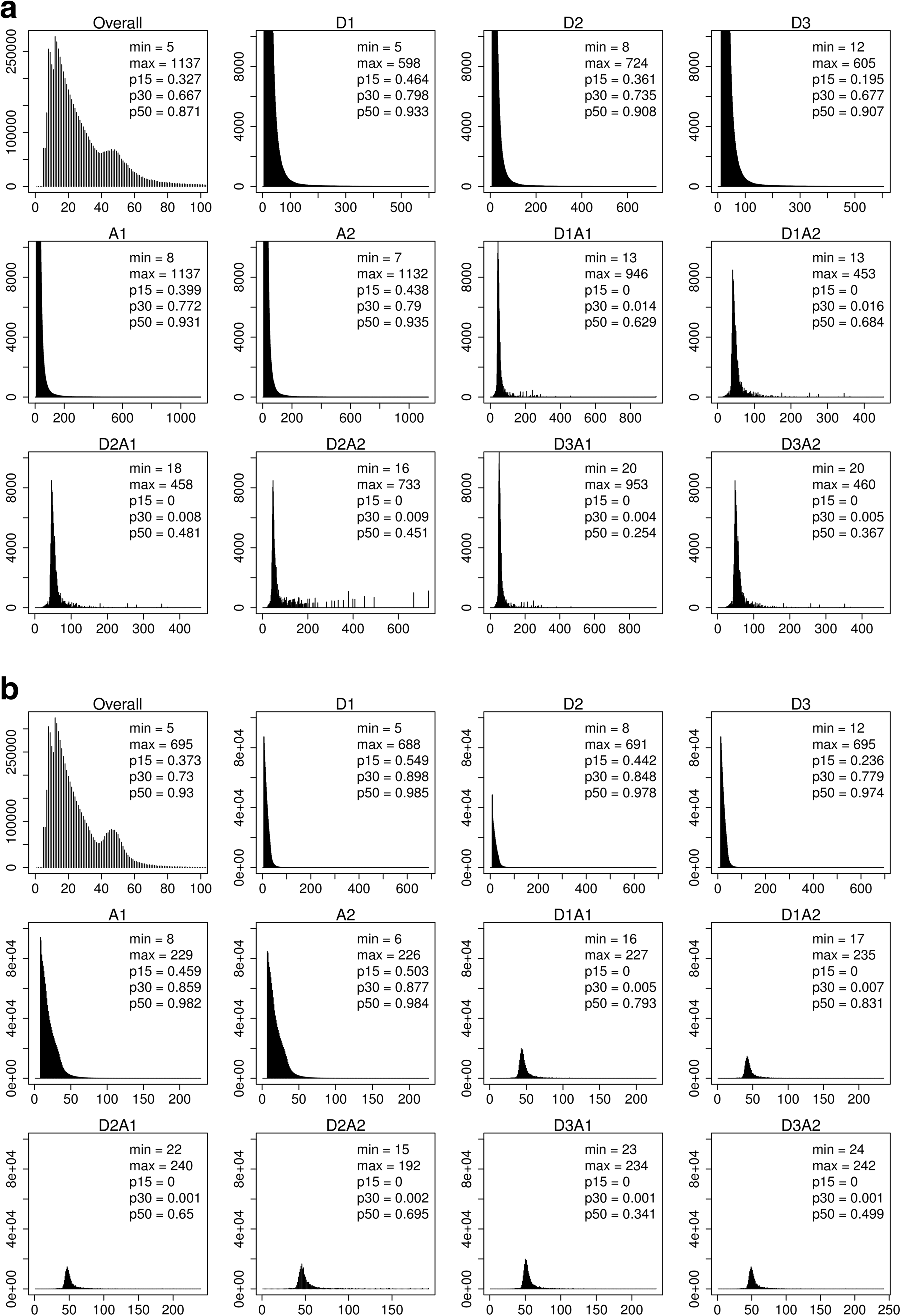

Supplement: Supplementary file 6 — Authors’ original file for figure 5 [file 40529_2012_17_MOESM6_ESM.tif]
